# Predominance of Other Pathogenic Bacteria among Presumptive Tuberculosis Cases Attending Tuberculosis Clinics in Mwanza, Tanzania: A Cross-Sectional Laboratory-Based Study

**DOI:** 10.3390/microorganisms10040703

**Published:** 2022-03-25

**Authors:** Florencia S. Buchera, Vitus Silago, Geofrey Japhet, Conjester I. Mtemisika, Prisca Damiano, Helmut A. Nyawale, Martha F. Mushi, Mariam M. Mirambo, Jeremiah Seni, Stephen E. Mshana

**Affiliations:** 1 Department of Microbiology and Immunology, Weill Bugando School of Medicine, Catholic University of Health and Allied Sciences, Mwanza 1464, Tanzania; florenciabuchiara@gmail.com (F.S.B.); priscadamian77@gmail.com (P.D.); helmutny@yahoo.com (H.A.N.); mushimartha@gmail.com (M.F.M.); mmmirambo@gmail.com (M.M.M.); senijj80@gmail.com (J.S.); stephen72mshana@gmail.com (S.E.M.); 2 Tuberculosis Section, Central Pathology Laboratory, Bugando Medical Centre, Mwanza 1370, Tanzania; geoojaph@gmail.com; 3 Central Pathology Laboratory, Department of Molecular Biology, Bugando Medical Centre, Mwanza 1370, Tanzania; conjestermtemisika@yahoo.com

**Keywords:** co-infections, *Mycobacterium tuberculosis* complex, other pathogenic bacteria, tuberculosis, Mwanza

## Abstract

This study was designed to determine the prevalence and co-infection of *Mycobacterium tuberculosis* and other pathogenic bacteria among presumptive cases of tuberculosis (TB) at selected hospitals in Mwanza, Tanzania. GeneXpert and conventional bacteriological culture and sensitivity were used for the detection of TB and other pathogenic bacteria, respectively. STATA version 13.0 was used for data analysis. The median (IQR) age of participants was 33 (19–51) years with males forming more than half (i.e., 59% (158/264)) of the participants. Microscopically, 29.5% (78/264) of the patients had polymorphonuclear leucocytes in the sputum samples. Approximately 7.2% (19/264), 16.3% (43/264), and 1.1% (3/264) of participants had TB, other pathogenic bacteria, and co-infections, respectively. One sample had growth of two other bacteria, resulting in a total of 44 isolated bacteria with the predominance of Gram-negative bacteria at 75.0% (33/44). The predominant species isolated was the *Klebsiella pneumoniae* complex at 52.3% (23/44). Overall, 27.3% (9/33) of GNB were resistant to third-generation cephalosporins, while Gram-positive bacteria were more resistant to erythromycin at 63.6% (7/11). Good quality sputa had a significantly higher yield of pathogenic bacteria than poor quality sputa (37.2% vs. 7.5%, *p* < 0.001). Presumptive TB cases were predominantly infected with other pathogenic bacteria than *M. tuberculosis*. Therefore, other pathogenic bacteria should be considered when attending presumptive TB cases to ensure favorable treatment outcomes.

## 1. Introduction

Tuberculosis (TB), a communicable disease caused by *Mycobacterium tuberculosis* complex, is a major cause of ill health and is ranked among the top 10 causes of death globally [1]. In Tanzania, a 2020 WHO report estimated the burden of TB at 237 cases per 100,000 population, accounting for 20 deaths per 100,000 cases among HIV-positive people and 35 deaths per 100,000 cases among HIV-negative people [1]. This is because deaths among HIV-positive TB cases are classified as HIV deaths, therefore underestimating the prevalence of death from TB among this population [1]. Tanzania was listed in a group of 30 countries with a high burden of TB; however, it is currently listed among the seven countries from its group to reach the 2020 milestone [1].

Currently, different studies have documented other bacterial infections as well as co-infection of TB and other bacteria among presumptive TB cases [2,3,4,5]. For example, a study by Arora et al. in India reported a case of co-infection of TB and *Klebsiella* spp. that was successful treated by meropenem only in a 55 year old male alcohol abuser who presented with fever, productive cough, and exertional breathlessness [2]. Another study in Cambodia by Attia et al. reported a prevalence of 30% TB, 44% other bacteria, and 9% co-infection of TB and other bacteria among presumptive TB cases [4]. Furthermore, this study reported that *Klebsiella* spp. (20%) and *Pseudomonas* spp. (15%) were predominantly isolated [4].

The prevalence of TB ranges from 1% to 34% among different populations in Mwanza, Tanzania [6,7,8,9,10]. In particular, the prevalence of laboratory-confirmed TB ranges from 5.2% to 33.5% among symptomatic TB cases [6,8,9], whereas the prevalence ranges from 1.3% to 3.9% among the asymptomatic population [7,10]. Although information on the prevalence of TB is well documented in this setting, the information on infection and co-infection of TB and other pathogenic bacteria among presumptive TB cases is not clearly known. Lack of this information leads to delayed diagnosis and inadequate treatment resulting in prolonged morbidity and increased associated mortality. Therefore, we designed this study to investigate the prevalence and co-infection of TB and other pathogenic bacteria among presumptive TB cases attending TB clinics in Mwanza, Tanzania.

## 2. Materials and Methods

### 2.1. Study Design, Population, Duration, and Setting

This cross-sectional laboratory-based study involved sputum samples collected from presumptive TB cases attending TB clinics at Sengerema Designated District Hospital (SDDH), Sekou Toure Regional Referral Hospital (SRRH), and Bugando Medical Centre (BMC) between June and August 2021. A study from the same setting, Mwanza [11], with a prevalence of 20.4%, was used to obtain a minimum sample size of 250 sputa using the Kish–Leslie formula [12].

### 2.2. Enrollment of Participants, Data, and Sample Collections

Participants were serially enrolled after the receipt of respective samples accompanied with laboratory request forms for diagnosis of TB. After the receipt of samples in the TB laboratory sections of the SDDH, SRRH, and BMC, participants were followed up in the respective clinics for data collection using a standardized questionnaire.

### 2.3. Laboratory Procedures

#### 2.3.1. Gram Staining of Sputum Samples for Assessing Sample Quality

The quality of sputa was assessed using Bartlett et al.’s criteria [13]. A light microscope under a low-power field (×10 objective lens) was used to look for: (1) the number of neutrophils per low-power field; (2) the presence of mucus strands; (3) the number of squamous epithelial cells per low-power field. Neutrophils were identified as small multilobed nucleus with 3–5 lobes joined together, while squamous epithelial cells were identified as cuboidal, flat, and sheet-like in appearance with single central nucleus. A sum of neutrophils/LPF: <10 score = 0, 10–25 score = +1, and >25 score = +2; mucus present score = +1; epithelial cells/LPF: 10–25 score = −1 and a >25 score = −2 was used to indicate “active inflammation” or “contamination”. A score of +1 or more indicated active inflammation, while a score of 0 or less indicated no inflammation or contamination with saliva.

#### 2.3.2. Culture Procedures for Isolation of Other Pathogenic Bacteria from Presumptive TB Cases

For isolation of other pathogenic bacteria, sputum samples were directly inoculated on chocolate agar (CA), 5% sheep blood agar (SBA; HiMedia, India), and MacConkey agar (MCA; HiMedia, India) plates, which were incubated in ambient air (SBA and MCA plates) and candle jar (CA plates) at 37 °C for 24 h (BA and MCA plates) and 48 h (CA plates). Bacteria growths were interpreted basing on colony morphologies (e.g., size and color) and characteristics (e.g., hemolysis on SBA and lactose fermentation on MCA) on respective culture plates [14].

#### 2.3.3. Biochemical Identification Testing of Other Pathogenic Bacteria

Gram staining was used for categorizing bacteria into Gram-negative and Gram-positive. Identification of Gram-negative bacteria included acid and CO_2_ production from sugars fermentation and production of H_2_S using triple sugar iron (TSI) agar; production of H_2_S and indole and motility using sulfur indole motility (SIM) agar; utilization of citrate as the sole source of carbohydrate using Simmons citrate agar; production of urease enzyme using Christensen urea agar; oxidase test [14]. Whereas for the identification of Gram-positive bacteria, catalase, coagulase, and DNase agar were used for identification and confirmation of *S. aureus*; type of hemolysis on SBA, catalase, bacitracin disk, trimethoprim-sulfamethoxazole disk, optochin disk, and bile aesculin tests were used for identification of *Streptococcus* spp. and *Enterococcus* spp. [14].

#### 2.3.4. Antibiotics Susceptibility Testing

For antibiotics susceptibility testing (AST), the disk diffusion technique by Kirby–Bauer was employed. Briefly, a bacterium suspension was prepared on sterile 0.9% normal saline, and the turbidity was adjusted to a 0.5 McFarland standard turbidity using a DensiCheck machine (bioMériux, Germany). Each bacterium suspension was then inoculated on the entire surface of Mueller Hinton agar (MHA; HiMedia, India) using sterile bacteriological cotton swab (Improswab; Guangzhou, China). Within 15 min of MHA inoculation with test bacterium, antibiotics disks were seeded. For Gram-negative bacteria, ampicillin 10 μg (AMP; Oxoid, UK), azithromycin 15 μg (AZM; Oxoid, UK), trimethoprim–sulfamethoxazole 25 μg (SXT; Oxoid, UK), tetracycline 30 μg (TE; Oxoid, UK), gentamicin 10 μg (CN; Oxoid, UK), ciprofloxacin 5 μg (CIP; Oxoid, UK), amoxicillin–clavulanic acid 10/20 μg (AMC; Oxoid, UK), ceftriaxone 30 μg (CRO; Oxoid, UK), piperacillin–tazobactam 100/10 μg (TZP; Oxoid, UK), amikacin 30 μg (AK; Oxoid, UK), and meropenem 10 μg (MEM; Oxoid, UK) disks were seeded. A combination disk method (CDM) using cephalosporins (i.e., cefotaxime 30 μg and ceftazidime 30 μg) with and without clavulanic acid was used for phenotypic confirmation of ESBL production among Gram-negative bacteria with resistance to ceftriaxone as recommended by the Clinical and Laboratory Standards Institute (CLSI) [15]. For Gram-positive bacteria, 10 units of penicillin, azithromycin 15 μg (AZM; Oxoid, UK), trimethoprim–sulfamethoxazole 1.25/23.75 μg (STX; Oxoid, UK), tetracycline 30 μg (TE; Oxoid, UK), gentamicin 10 μg (CN; Oxoid, UK), ciprofloxacin 5 μg (CIP; Oxoid, UK), erythromycin 15 μg (E; Oxoid, UK), cefoxitin 30 μg (FOX; Oxoid, UK; for *S. aureus* only), clindamycin 2 μg (CD; Oxoid, UK), and vancomycin (HiMedia, India) were seeded. Isolates of *S. aureus* with a zone of inhibition of ≤21 mm for cefoxitin were considered methicillin-resistant *S. aureus* (MRSA) [15].

Additionally, an agar dilution test on MHA plates was performed to determine susceptibility and minimum inhibitory concentrations (MICs; 2, 4, 8, 16, 32, 64, and 128 μg/mL) of Gram-negative bacteria against polymyxin B (POLY-MxB; Bharat serums and vaccines limited, India) and *S. aureus* against vancomycin (Vancolin-500; Lincoln Pharmaceuticals Limited, India). All AST results by disk diffusion and agar dilution tests were interpreted as per the Clinical and Laboratory Standards Institute (CLSI) guidelines of 2020 [15].

#### 2.3.5. GeneXpert for Detection of TB

TB was diagnosed by the GeneXpert test that detects the presence of the *M. tuberculosis* complex and resistance to rifampin (RIF). Sputum sample was mixed with sample reagent in a ratio of 2:1 to liquefy for 15 min. Then, 2 mL of the mixture was transferred to a cartridge, and the cartridge was then inserted into the GeneXpert system to begin detection [16].

#### 2.3.6. Quality Control

*E. coli* ATCC 25922 and *S. aureus* ATCC 25923 were used as control organisms.

#### 2.3.7. Storage of Other Pathogenic Bacteria

All other pathogenic bacteria isolated in this study were stored in vials containing 20% glycerol in brain heart infusion (BHI) broth and archived in a −40 °C forty deep freezer for retrieval whenever necessary.

### 2.4. Data Management and Analysis

Laboratory data were recorded in laboratory logbook and then entered into Microsoft Excel merged with other sociodemographic and clinical data for cleaning and coding. STATA 13.0 was used for data analysis. Categorical data are presented in percentages and fractions, while continuous data are presented as the mean (±SD) and median (interquartile range: IQR). Pearson’s chi-square and Fisher’s exact tests were used for testing association between the categorical outcome and the categorical predictors. A *p*-value of less than 0.05 at a 95% confidence interval (95%CI) was considered statistically significance.

### 2.5. Ethical Clearance Considerations

Ethical clearance to conduct this study was requested from the joint Catholic University of Health and Allied Sciences (CUHAS) and the Bugando Medical Centre (BMC) Research Ethics and Review Committee. A certificate number CREC: 1888/2021 was offered. Permission to conduct this study was sought from the Director General and the Director of Laboratory Services of BMC and the Medical Officer In-Charge and Laboratory Manager of SRRH and SDDH.

## 3. Results

### 3.1. Sociodemographic and Clinical Characteristics of Study Participants

A total of 264 sputum samples were collected during the study period. The mean (±SD) age of patients whose samples were enrolled was 36.6 (±19.7) years. The majority of sputum samples were collected from the male gender (i.e., 59.8% (158/264)) and outpatients (96.6% (255/264)). On the other hand, approximately 20.1% (53/264) and 2.7% (7/264) of patients had a history of fever and antibiotics use in the past 3 months before being enrolled in this study. Approximately 13.6% (36/264) of patients had chronic disease conditions of which the majority had HIV 83.3% (30/36). Moreover, approximately 3.8% (10/264) of patients had a history of TB infection. All patients enrolled had at least one symptom (e.g., fever, cough, and breathlessness) of respiratory tract infection (Table 1).

### 3.2. Microscope, Culture, and GeneXpert Results

**Figure 1:**
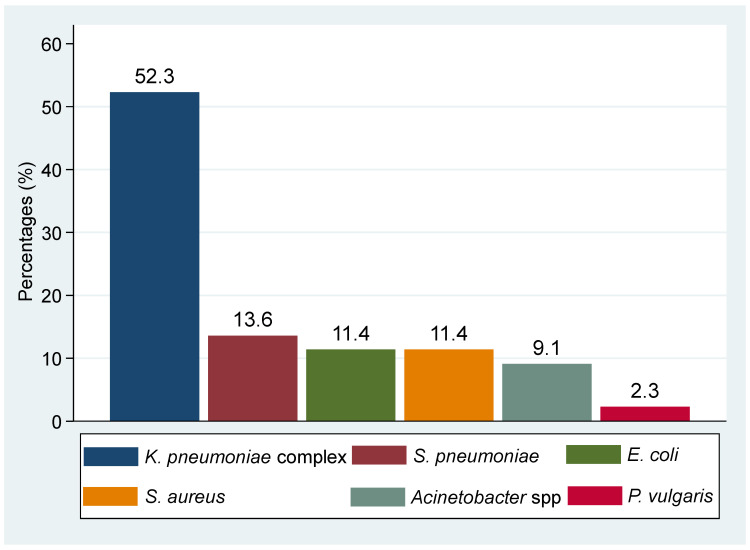
Proportions of other pathogenic bacteria isolated from presumptive TB cases.

**Figure 2:**
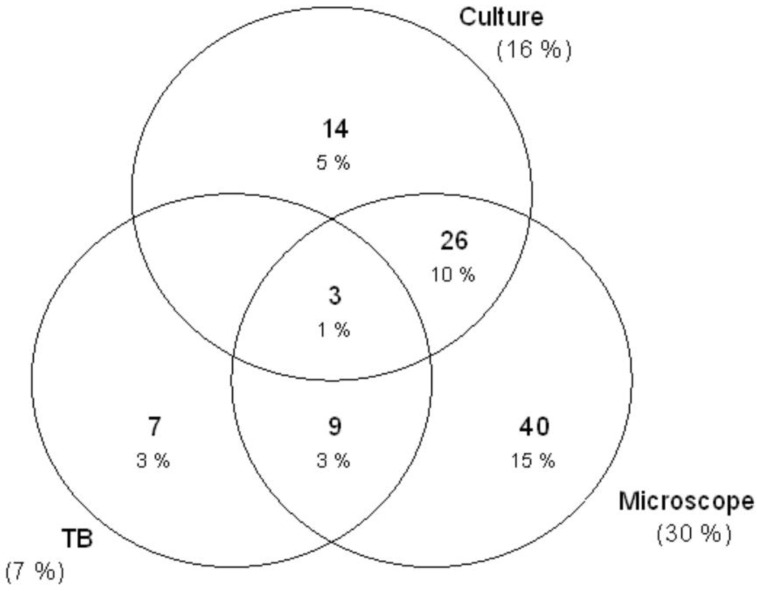
Venn diagram showing the prediction of microscope for culture and TB positive results and co-infection of TB and other pathogenic bacteria. Three participants with active inflammation had both *M. tuberculosis* complex and other pathogenic bacterial infections (center); 29 (3 + 26) participants with active inflammation had infection with other pathogenic bacteria only; 12 (3 + 9) participants with active inflammation had infection with *M. tuberculosis* complex only.

Nearly one-third (i.e., 29.5% (78/264)) of sputum samples showed active inflammation microscopically. Approximately 7.2% (19/264) had positive GeneXpert results for pulmonary TB of which 10.5% (2/19) had a previous history of TB. On the other hand, approximately 16.3% (43/264) had a positive culture with other pathogenic bacteria. Presumptive TB cases were predominantly infected with other pathogenic bacteria than *M. tuberculosis,* although the difference was not statistically significant (16.3% vs. 7.2%, *p* = 0.1669). One sample had growth of two other bacteria making a total of 44 isolated bacteria with the predominance of Gram-negative bacteria at 75.0% (33/44). The predominant species isolated was the *Klebsiella pneumoniae* complex (52.3% (23/44)), followed by *S. pneumoniae* (13.6% (6/44)), *E. coli* (11.4% (5/44)), *S. aureus* (11.4% (5/44)), *Acinetobacter* spp. (9.1% (4/44)), and *P. vulgaris* (2.3% (1/44)) (Figure 1). Whereas co-infection of TB and other pathogenic bacteria was observed in 1.1% (3/264), two co-infections with *K. pneumoniae* complex, and one co-infection with *S. aureus* (Figure 2).

### 3.3. Percentages Resistance of Other Pathogenic Bacteria Isolated from Presumptive TB Cases

Gram-negative bacteria showed high resistance to ampicillin (96.9%). In specific, four Gram-negative bacteria, including, *Acinetobacter* spp. (*n* = 2), *P. vulgaris* (*n* = 1), and *Klebsiella pneumoniae* complex (*n* = 1) had polymyxin-B MIC as high as 128 μg/mL. Overall, 27.3% (9/33) of the Gram-negative bacteria showed resistance to third-generation cephalosporins (i.e., ceftriaxone), of which 33.3% (3/9; two *E. coli* and one *P. vulgaris*) were phenotypically confirmed as ESBL producers. On the other hand, Gram-positive bacteria showed high resistance towards trimethoprim–sulfamethoxazole (90.0%) and high sensitivity towards vancomycin (100%) and linezolid (100%). Specifically, 1 (20%) out of 5 *S. aureus* showed resistance to cefoxitin, hence, methicillin-resistant *S. aureus* (MRSA), while all *S. pneumoniae* (*n* = 6) were susceptible to penicillin (Table 2).

### 3.4. Factors Associated with a Culture Positive with Other Pathogenic Bacteria among Presumptive TB Cases

**Figure 3:**
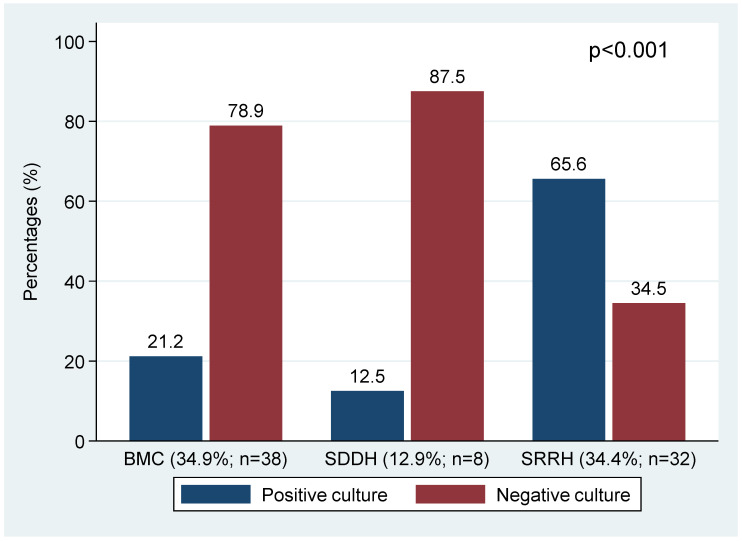
The prevalence of active inflammation per hospital and the proportions of cultures positive for other pathogenic bacteria among presumptive TB cases with active inflammation under microscope.

Using Pearson’s chi-square test, other pathogenic bacteria were significantly more present in sputum samples at SRRH than BMC and SDDH (29.1% vs. 12.8% and 3.2%, *p* < 0.001), and sputum samples with active inflammation significantly predicted a culture positive with other pathogenic bacteria (37.2% vs. 7.5%, *p* < 0.001) (Table 3 and Figure 3).

### 3.5. Factors Associated with Positive TB Results among Presumptive TB Cases

Using Pearson’s chi square test, other pathogenic bacteria were significantly more present in sputum samples at SRRH than BMC and SDDH (15.1% vs. 4.6% and 0%, *p* < 0.001), in sputum samples with active inflammation significantly predicted a culture positive with other pathogenic bacteria (15.4% vs. 3.8%, *p* < 0.001) (Table 4).

## 4. Discussion

This study enrolled participants who presented with at least one clinical symptom of lower respiratory tract infection suggestive of TB infection. The majority of participants enrolled were outpatients similar to a previous study from the same setting that investigated community-acquired pneumonia [11]. Moreover, the majority of participants were female, and nearly one-half were enrolled in a tertiary healthcare facility, BMC. Of those, only seven out of the 264 participants were currently on antibiotics use; five were on amoxicillin, an antibiotic recommended for treatment of typical community-acquired pneumonia in outpatients [17]. Of those participants reported having chronic diseases (12.5%), the majority (83.3%) had HIV. Further, approximately 3.8% (10 cases) reported being previously infected with TB, suggesting recurrence of TB infection. These findings reiterate the fact that people with chronic diseases and those with a previous history of TB remain at increased risk of contracting TB. Therefore, a need to strengthen health systems to timely identify them and provide appropriate management is emphasized in this study.

The prevalence of positive TB among clinically presumed cases in our study was 7.2% by GeneXpert. This prevalence was lower compared to studies conducted before 2016 and almost similar to studies conducted after 2016 from the same setting [6,8,9,10]. This indicates that, as reported by the WHO Global Tuberculosis Report 2020 [1], Tanzania is among seven countries among a list 30 countries with a high TB burden to reach the 2020 milestone. Moreover, two positive TB cases in our study had a previous history of TB; however, we did not establish a timeline between the previous and current episodes. This was also reported previous from the same setting [18]. Although, we did not perform a culture to ensure the viability of the two cases to be confident to report relapse of TB. TB patients may continue to expectorate dead *M. tuberculosis* on their sputum for some time [19]. Nucleic acid amplification, i.e., GeneXpert used for detection of TB, could not distinguish dead from live *M. tuberculosis* [20].

In the current study, other pathogenic bacteria were detected in 16.3% among presumptive TB cases. The prevalence of other pathogenic bacteria in our study was lower compared to a similar study which reported a prevalence of 44% of other pathogenic bacteria in Cambodia [4]. The difference could be attributed to the fact that in the current study, we enrolled only presumptive TB cases, while in the Cambodian study, they enrolled TB cases and other pathogenic bacterial cases. In line with previous studies in the same setting [11] and elsewhere [4], Gram-negative bacteria, predominantly *K. pneumoniae* complex, was a frequently encountered pathogen. We also isolated *S. pneumoniae*, a common cause of pneumococcal diseases, in community-acquired pneumonia [21,22] from six presumptive TB cases. Other pathogenic bacteria, including *E. coli*, *S. aureus*, *Acinetobacter* spp., and *P. vulgaris,* were also isolated. We speculate that participants with neither TB infection nor other pathogenic bacteria infection may be infected with fungal or viral pathogens and, hence, there is a need to extend our diagnostic services to be able to screen other pathogens routinely [23]. Moreover, antibiotics exposure might have reduced the sensitivity of culture for other bacteria as reported previously [24,25]. This was evidenced in this study whereby seven sputum samples from patients with prior antibiotics use had no bacteria yield despite three samples having active inflammation under microscope.

The prevalence (1.1%) of TB and other bacterial co-infection in our study was low compared to a similar study from Cambodia that reported a prevalence of 9% [4]. The difference may be attributed to two factors: first, the majority of our study participants were young (mean age: 36.6 ± 19.7 years), while the majority of the Cambodian study were elders (median age: 52 (37–64) years); second was the low prevalence of TB in our study compared to the high prevalence of TB in the Cambodian study (7.2% vs. 29%). As reported previous [26,27], older age and pulmonary TB infections may predispose individuals’ respiratory tracts to infections with other pathogenic bacteria.

We observed that Gram-negative bacteria were more resistant to ampicillin but less resistant to polymyxin-B, amikacin, and meropenem. Similar findings were reported previously from the same setting, that Gram-negative bacteria causing community-acquired pneumonia were more resistant to ampicillin but less resistant to meropenem [11]. Community access to penicillins (i.e., ampicillin) and the nature of the prescription (e.g., polymyxin-B, amikacin, or meropenem are prescribed after culture and sensitivity) are major drivers of higher bacteria resistance to ampicillin but less resistance to polymyxin-B, amikacin, and meropenem in this setting. Moreover, approximately one-third of Gram-negative bacteria were resistant to third-generation cephalosporins, i.e., ceftriaxone of which one-third were confirmed as ESBL producers phenotypically. On the other hand, Gram-positive bacteria were more resistant to trimethoprim–sulfamethoxazole and erythromycin but not resistant to vancomycin and linezolid. This could be due to the fact that community have access to trimethoprim–sulfamethoxazole and erythromycin but no access to vancomycin and linezolid. Moreover, one-fifth of *S. aureus* was confirmed as methicillin-resistant *S. aureus* (MRSA). Generally, recovery of MRSA and ESBL producing Gram-negative bacteria in our study suggests that multidrug resistance (MDR) pathogenic bacteria are circulating in the community.

Good quality sputum samples had significantly more yield of other pathogenic bacteria and TB/GeneXpert positive results than poor quality sputum as reported previously [11]. The predominance of polymorphonuclear (PMN) cells over squamous cells indicates the active inflammation of the respiratory tract, and it differentiates true pathogens from oropharyngeal contamination [28,29]. Therefore, Gram-stained sputum samples showing active inflammation under microscope significantly increase isolation of respiratory pathogens and detection of *M. tuberculosis* complex in sputum samples.

## 5. Conclusions

Presumptive TB cases are predominantly infected with other pathogenic bacteria than *M. tuberculosis* complex. Therefore, other pathogenic bacteria should be considered when attending presumptive TB cases. High-quality sputum had significantly more yield of other pathogenic bacteria and positive TB than poor quality sputum. This emphasizes the importance of providing proper instructions to the patients to ensure high-quality sputum specimens are obtained for microbiological investigations.

## Figures and Tables

**Table 1:** Sociodemographic and clinical characteristics of the study participants.

Characteristics		Frequency (*n*)/mean (±SD)	Percentages (%)
Mean (±SD) age in years		36.6 (±19.7)	-
Gender	Female	106	40.2
	Male	158	59.8
Healthcare of enrolment	BMC	109	41.3
	SRRH	93	35.2
	SDDH	62	23.5
Patient category	Inpatient	9	3.4
	Outpatient	255	96.6
History of fever over past 3 months	Yes	53	20.1
	No	211	79.9
History of antibiotic use	Yes	7	2.7
	No	257	97.3
Type of antibiotic used (*N* = 7)	Amoxicillin	5	71.4
	Other	2	28.6
Participant with chronic disease	Yes	33	12.5
	No	231	87.5
Type of chronic disease (*N* = 36) *	HIV	30	83.3
	Other **	6	16.7
Current symptom of upper respiratory tract	Yes	264	100.0
	No	0	0.0
Previous history of TB	Yes	10	3.8
	No	254	96.2

* Total types of chronic diseases = 36; three participants had two chronic diseases each (HV and diabetes mellitus (*n* = 2) and HIV and cancer (*n* = 1). ** Other: cancer (*n* = 3), diabetes mellitus (DM; *n* = 2), and asthma (*n* = 1).

**Table 2:** Percentages of resistance of other pathogenic bacteria isolated from presumptive TB cases.

Antibiotic Agents Tested	Gram-Negative Bacteria *n* (%)	Gram-Positive Bacteria *n* (%)
AMP	32 (96.9)	NA
SXT	12 (36.4)	10 (90.9)
TE	13 (39.4)	6 (54.5)
CN	8 (24.2)	3 (27.3)
CIP	15 (45.4)	4 (36.4)
TZP	13 (39.4)	NA
CRO	9 (27.3)	NA
MEM	3 (9.1)	NA
AK	2 (6.1)	NA
Polymyxin-B	6 (18.2)	NA
E	NA	7 (63.6)
CD	NA	6 (54.5)
FOX (*S. aureus, n* = 5)	NA	1 (20.0)
P (*S. pneumoniae, n* = 6)	NA	0 (0.0)
VA	NA	0 (0.0)
LZD	NA	0 (0.0)

AMP = ampicillin; SXT = trimethoprim–sulfamethoxazole; TE = tetracycline; CN = gentamicin; CIP = ciprofloxacin; TZP = piperacillin–tazobactam; CRO = ceftriaxone; MEM = meropenem; AK = amikacin; E = erythromycin; CD = clindamycin; FOX = cefoxitin; NA = not applicable.

**Table 3:** Factors associated with a culture positive with other pathogenic bacteria among presumptive TB cases.

Characteristics		Culture Results		Pearson Chi^2^ Test	
		Negative *n* (%)	Positive *n* (%)	X^2^	*p*-Value
Gender	Female	87 (82.1)	19 (17.9)		
	Male	134 (84.8)	24 (15.2)	0.3479	0.555
Healthcare facility of enrolment	BMC	95 (87.2)	14 (12.8)		
	SRRH	66 (70.9)	27 (29.1)		
	SDDH	60 (96.8)	2 (3.2)	19.7845	<0.001
History of fever	No	173 (81.9)	34 (18.1)		
	Yes	48 (90.6)	5 (9.4)	2.2847	0.131
History of antibiotic use	No	214 (83.3)	43 (16.7)		
	Yes	7 (100.0)	0 (0.0)	1.3991	0.237
Chronic disease condition	No	196 (84.6)	35 (15.2)		
	Yes	25 (75.8)	8 (24.2)	1.7502	0.186
Microscope inflammation	Contamination	172 (92.5)	14 (7.5)		
	Inflammation	49 (62.8)	29 (37.2)	35.4386	<0.001
TB status	Negative	205 (83.7)	40 (16.3)		
	Negative	16 (84.2)	3 (15.8)	0.0037	0.951

BMC = Bugando Medical Centre; SDDH = Sengerema Designated District Hospital; SRRH = Sekou Toure Regional Referral Hospital.

**Table 4:** Factors associated with positive TB results among presumptive TB cases.

Characteristics		TB/GeneXpert Results		Pearson Chi^2^ Test	
		Negative *n* (%)	Positive *n* (%)	X^2^	*p*-Value
Gender	Female	101 (95.3)	5 (4.7)		
	Male	144 (91.1)	14 (8.9)	1.6309	0.202
Healthcare facility of enrolment	BMC	104 (95.4)	5 (4.6)		
	SRRH	79 (84.9)	14 (15.1)		
	SDDH	62 (100.0)	0 (0.0)	14.5150	0.001
History of fever	No	196 (92.9)	15 (7.1)		
	Yes	49 (92.5)	4 (7.6)	-	1.000 *
History of antibiotic use	No	239 (93.0)	18 (7.0)		
	Yes	6 (84.7)	1 (14.3)	-	0.411 *
Chronic disease condition	No	212 (91.8)	19 (8.2)		
	Yes	33 (100.0)	0 (0.0)	-	0.143 *
Microscope inflammation	Contamination	179 (96.2)	7 (3.8)		
	Inflammation	66 (84.6)	12 (15.4)	11.1120	0.001

BMC = Bugando Medical Centre; SDDH = Sengerema Designated District Hospital; SRRH = Sekou Toure Regional Referral Hospital. * Fisher’s exact test was used.

## Data Availability

The data presented in this study are available upon request from the corresponding author.
